# Supplementary figures and images for: Comprehensive review of safety in Experimental Human Pneumococcal Challenge
Source: PLoS One. 2023 May 4;18(5):e0284399. doi: 10.1371/journal.pone.0284399 (PMC10159102; doi:10.1371/journal.pone.0284399)

**S1 Figure: Timeline of EHPC studies 2011-2021**


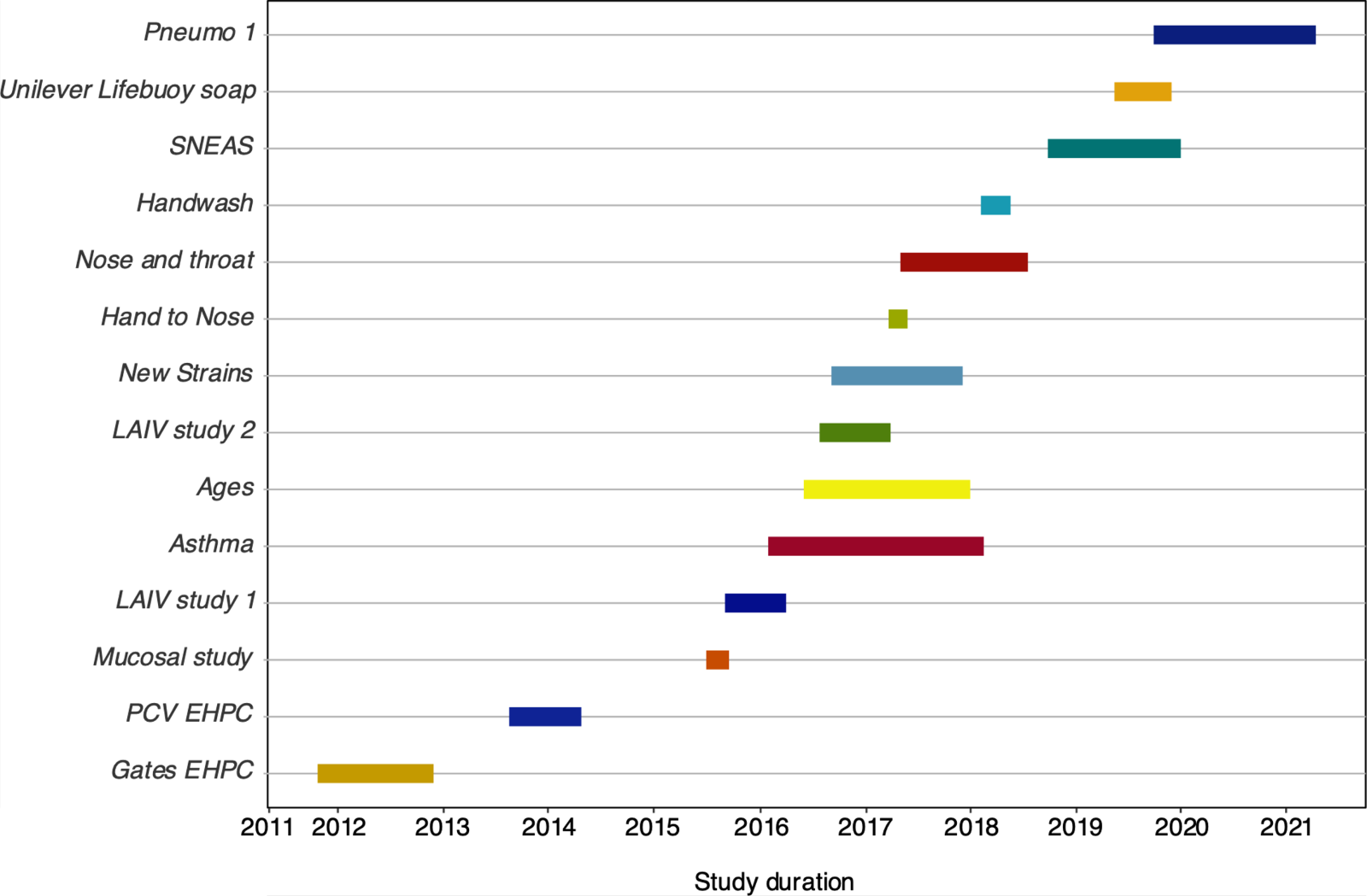

Supplement: S1 Fig — Gantt chart ranging from 2011 to 2022 with each EHPC study represented by a separate colour. Study commencement was defined as the time of first participant consent and the study end was defined as the last participant visit. (DOCX) [file pone.0284399.s001.docx]

## **S2 Figure: Heatmap of reported potential pneumococcal symptoms in EHPC studies**


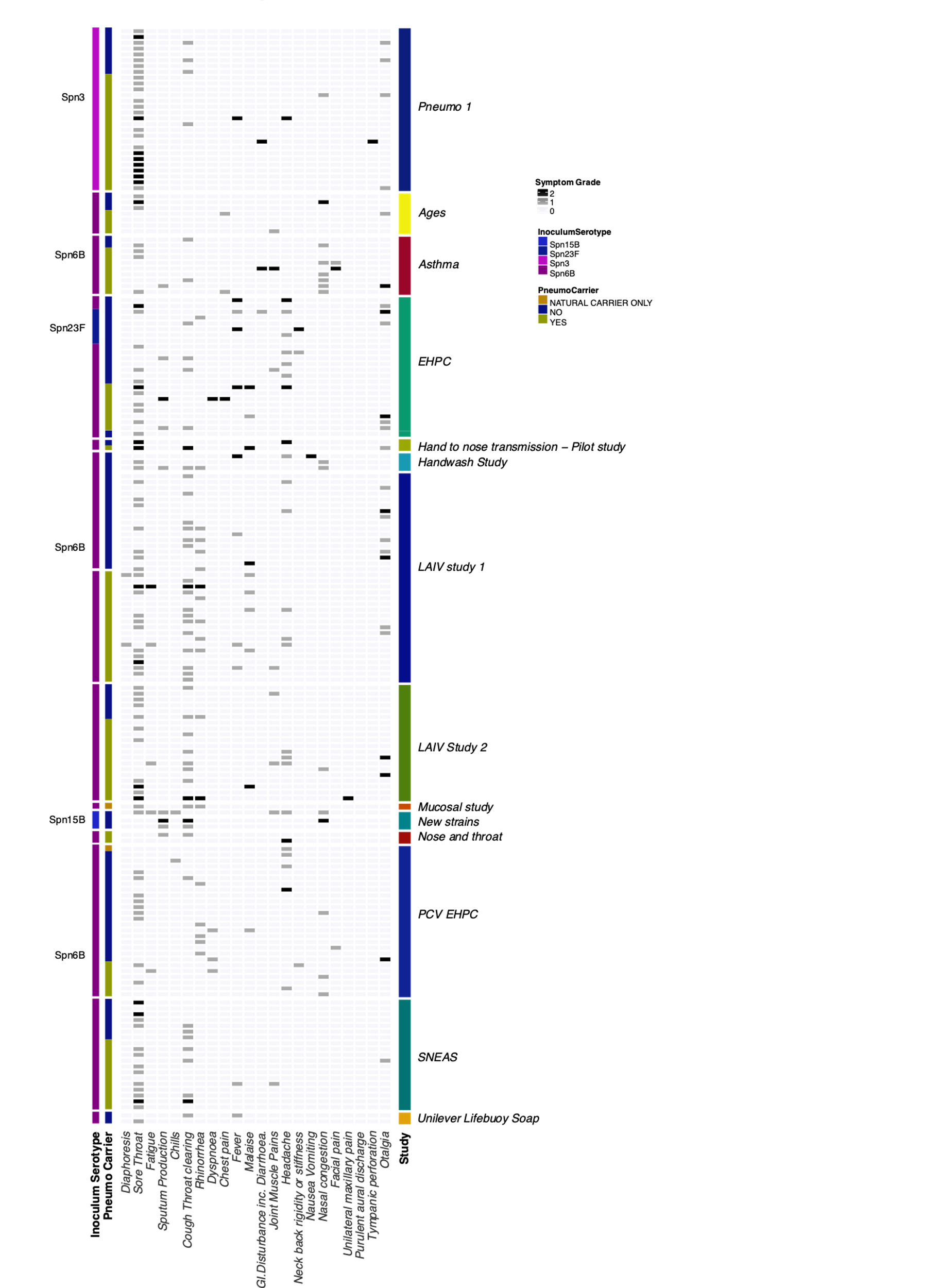

Supplement: S2 Fig — All included studies are shown in different colours with each individual reporting symptoms on a separate row. All reported symptoms are shown in individual columns. Grade 1 severity symptoms shown in grey and grade 2 in black. The pneumococcal colonised are in green and non-pneumococcal colonised in blue. Each inoculum serotype is shown in different colours (SPN6- green, SPN3- pink, SPN15B- dark green, SPN23F-blue). Sore throat is the most commonly reported symptom. (DOCX) [file pone.0284399.s002.docx]
