# Supplementary material for: Comprehensive review of safety in Experimental Human Pneumococcal Challenge
Source: PLoS One. 2023 May 4;18(5):e0284399. doi: 10.1371/journal.pone.0284399 (PMC10159102; doi:10.1371/journal.pone.0284399)
Supplement: S3 Fig — IM = 1 refers to the’ pipette- to-nose’ method of inoculation and IM = 2 refers to the ‘hand-to-nose method of inoculation. Effect sizes are reported as OR with 95% CI. The overall OR is shown in bold, and a dotted line included for comparison across studies. (DOCX) [file pone.0284399.s003.docx]

## **S3 Figure: Forest plot of all potentially pneumococcal related safety reviews across all included EHPC studies using a random effects model.**

**
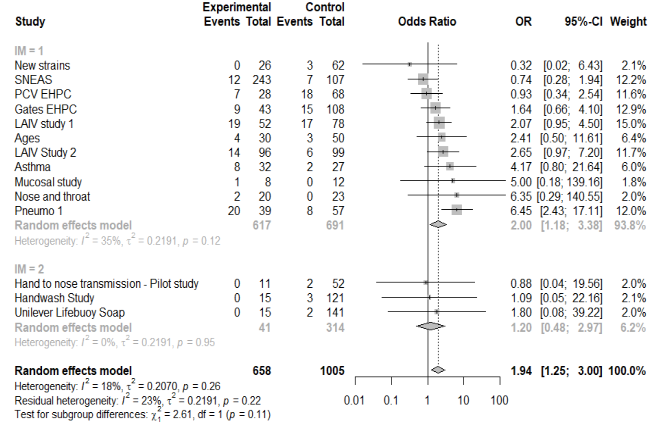
**
