# Supplementary material for: Comprehensive review of safety in Experimental Human Pneumococcal Challenge
Source: PLoS One. 2023 May 4;18(5):e0284399. doi: 10.1371/journal.pone.0284399 (PMC10159102; doi:10.1371/journal.pone.0284399)
Supplement: S1 Table — (DOCX) [file pone.0284399.s004.docx]

| **Inclusion criteria** | |
| --- | --- |
| Healthy young adults aged 18-50 years (inclusive)* | |
| Fluent spoken English | |
| Access to their own mobile telephone | |
| Capacity to give informed consent | |
| **Exclusion criteria** | |
| ***Research*** | Currently involved in another study (unless observational or non-interventional except for the EHPC bronchoscopy study) |
|  | Participant in a previous EHPC trial within the last 3 years |
| ***Pneumococcal colonisation*** | Current pneumococcal colonisation at screen* |
| ***Vaccine*** | Previous pneumococcal vaccination PPV23, PCV13 or PCV10 |
| ***Allergies*** | Allergic to penicillin (for PneumEx if the participant was not allergic to Clarithromycin in addition this was not an exclusion criteria) |
| ***Health history*** | Chronic ill health or higher risk of infection including, immunosuppressive history, diabetes, asthma* (on regular medication), recurrent otitis media or other respiratory disease |
|  | Medication that may affect the immune system or clotting e.g. steroids, inflammation altering (eg. nasal steroids, roacutane or aspirin) or disease-modifying anti-rheumatoid drugs |
|  | Recent antibiotics within the last 28 days or long term for known active chronic infection |
|  | Current illness or acute illness within 14 days prior to inoculation |
|  | Major pneumococcal illness during lifetime requiring hospitalisation |
|  | Other conditions considered by the clinical team as a concern for participant safety or integrity of the study |
| ***Direct caring role or close contact*** | With individuals at higher risk of infection: |
|  | Children under 5 years of age |
|  | Chronic ill health or immunosuppressed adults |
| ***Smokers*** | Current or ex-smoker (regular cigarettes, e-cigarettes / vaping, and regular smoking of recreational drugs) in the last 6 months |
|  | Previous significant smoking history – more than 20 cigarettes per day for 20 years or the equivalent (>20 pack years) |
| ***Women of child bearing age*** | Insufficient or ineffective birth control |
|  | Currently pregnant |
| ***History of current drug or alcohol abuse*** | Frequently drinking alcohol:  Men and women should not regularly drink > 3 units/day and 2 units/day respectively - assessed at the discretion of the clinician |
| ***Travel*** | Overseas travel planned in the follow up period |

**S1 Table: Inclusion and Exclusion criteria in Experimental Human Pneumococcal Challenge**

*unless otherwise specified in the study protocol
