# Supplementary material for: Comprehensive review of safety in Experimental Human Pneumococcal Challenge
Source: PLoS One. 2023 May 4;18(5):e0284399. doi: 10.1371/journal.pone.0284399 (PMC10159102; doi:10.1371/journal.pone.0284399)
Supplement: S2 Table — (DOCX) [file pone.0284399.s005.docx]

## **S2 Table: Search terms used to define pneumococcal symptoms**

| ***Streptococcus pneumoniae*** | |
| --- | --- |
|  | *‘Streptococcus pneumoniae’*  ‘Pneumococcus’  ‘Pneumococcal’  ‘Pneumococcal disease’  ‘Invasive pneumococcal disease’  ‘IPD’ |
| **Symptoms** | |
|  | ‘Symptoms’  ‘Symptom’  ‘Clinical syndromes’  ‘Clinical syndrome’  ‘Clinical presentation’  ‘Clinical features’ |
| **PHE expected disease syndromes due to *S.pneumoniae*** | |
| **Pneumonia** | |
|  | ‘Pneumonia’  ‘Community acquired pneumonia’  ‘Lung infection’ |
| **Meningitis** | |
|  | ‘Meningitis’  ‘Brain infection’ |
| **Sepsis** | |
|  | ‘Sepsis’  ‘Septicaemia’  ‘Bacteraemia’  ‘Bloodstream infection’ |
| **Acute otitis media** | |
|  | ‘Acute otitis media’  ‘Otitis media’  ‘Ear infections’ |
| **Sinusitis** | |
|  | ‘Sinusitis’  ‘Rhinosinusitis’  ‘Sinus infection’ |
