# Supplementary material for: Comprehensive review of safety in Experimental Human Pneumococcal Challenge
Source: PLoS One. 2023 May 4;18(5):e0284399. doi: 10.1371/journal.pone.0284399 (PMC10159102; doi:10.1371/journal.pone.0284399)
Supplement: S3 Table — (DOCX) [file pone.0284399.s006.docx]

## **S3 Table: Potential Pneumococcal symptoms expected by the most frequent pneumococcal disease syndromes as defined by UK HSA**

| **UK HSA pneumococcal disease syndrome** | **Clinical features identified from literature** |
| --- | --- |
| Pneumonia | Fever^1-10^  Cough^1-6,8-12^  Sputum production^1-5,7,8,10-12^  Chest pain^1,3-5,8-12^  Dyspnoea^1-4,6-9,11^  GI disturbance^1-4,8-10^  Chills^3,8,10,12^  Fatigue^3,11^  Headache^2,8,10^  Arthralgia/myalgia^3^  Haemopytsis^4,9,10^  Diaphoresis^4^  Confusion^7-10^  Sore throat^8,10^  Rhinorrhea^8^ |
| Meningitis | Headache^13-19^  Generalized or focal seizures^15-17,19-21^  Altered mental status^13,15-18,20,21^  Coma^16,20^  Neck/back rigidity or stiffness^15-19,21^  Fever^15-19^  Focal neurologic deficits^15,17-19,21^ e.g. aphasia, hemiparesis, cranial nerve palsies  Photophobia^18,19^  Nausea/vomiting^18,19^  Hearing loss^17^  Poor appetite^18^ |
| Septicaemia | Septic shock^22,23^  Fever^22^  Joint and muscle pains^22^  Nausea^22^  Malaise^22^  Vomiting^22^  Diarrhoea^22^ |
| Otitis media | Otalgia^24^ ^25,26^  Fever^24-26^  Tympanic perforation^24^  Purulent aural discharge^24^ |
| Sinusitis | Coloured nasal discharge^27^  Facial pain^27^  Facial pain on bending forward^28^  Unilateral maxillary pain^28^  Maxillary toothache^28,29^  Pus in the nasal cavity^28^  Nasal congestion^30,31^  Postnasal drainage/discharge^30^  Cough/throat clearing^30^  Malar tenderness/pain^30^  Frontal headache^30^  Hyposmia^32^  Purulent rhinorrhea^28^ |

References

1. Gentile JH, Sparo MD, Mercapide ME, Luna CM. Adult bacteremic pneumococcal pneumonia acquired in the community. *Medicina (Buenos Aires).* 2003;63(1):9-14.

2. Sopena N, Luisa Pedro-botet M, Sabrià M, García-Parés D, Reynaga E, García-Nuñez M. Comparative study of community-acquired pneumonia caused by Streptococcus pneumoniae, Legionella pneumophila or Chlamydia pneumoniae. *Scandinavian journal of infectious diseases.* 2004;36(5):330-334.

3. Beović B, Bonač B, Keše D, et al. Aetiology and clinical presentation of mild community-acquired bacterial pneumonia. *European Journal of Clinical Microbiology and Infectious Diseases.* 2003;22(10):584-591.

4. Bjarnason A, Westin J, Lindh M, et al. Incidence, etiology, and outcomes of community-acquired pneumonia: a population-based study. Paper presented at: Open forum infectious diseases2018.

5. Marrie TJ. Bacteraemic pneumococcal pneumonia: a continuously evolving disease. *Journal of infection.* 1992;24(3):247-255.

6. Watanakunakorn C, Bailey TA. Adult Bacteremic Pneumococcal Pneumonia in a Community Teaching Hospital, 1992-1996n: A Detailed Analysis of 108 Cases. *Archives of internal medicine.* 1997;157(17):1965-1971.

7. Pletz M, Von Baum H, Van der Linden M, et al. The burden of pneumococcal pneumonia–experience of the German competence network CAPNETZ. *Pneumologie.* 2012;66(08):470-475.

8. Hung Y-P, Wu C-J, Chen C-Z, et al. Comparisons of clinical characters in patients with pneumococcal and Legionella pneumonia. *Journal of Microbiology, Immunology and Infection.* 2010;43(3):215-221.

9. Lippmann ML, Goldberg SK, Walkenstein MD, Herring W, Gordon M. Bacteremic pneumococcal pneumonia: a community hospital experience. *Chest.* 1995;108(6):1608-1613.

10. Woodhead M, Macfarlane J. Comparative clinical and laboratory features of legionella with pneumococcal and mycoplasma pneumonias. *British journal of diseases of the chest.* 1987;81:133-139.

11. Brandenburg JA, Marrie TJ, Coley CM, et al. Clinical presentation, processes and outcomes of care for patients with pneumococcal pneumonia. *Journal of general internal medicine.* 2000;15(9):638-646.

12. Bohte R, Hermans J, Van den Broek P. Early recognition of Streptococcus pneumoniae in patients with community-acquired pneumonia. *European Journal of Clinical Microbiology and Infectious Diseases.* 1996;15(3):201-205.

13. Vestergaard HH, Larsen L, Brandt C, et al. Normocellular Community-Acquired Bacterial Meningitis in Adults: A Nationwide Population-Based Case Series. *Annals of Emergency Medicine.* 2021;77(1):11-18.

14. Østergaard C, Høiby N, Bossen Konradsen H, Samuelsson S. Prehospital diagnostic and therapeutic management of otogenic Streptococcus pneumoniae meningitis. *Scandinavian journal of infectious diseases.* 2006;38(3):172-180.

15. Østergaard C, Konradsen HB, Samuelsson S. Clinical presentation and prognostic factors of Streptococcus pneumonia e meningitis according to the focus of infection. *BMC infectious diseases.* 2005;5(1):1-11.

16. Van de Beek D, De Gans J, Spanjaard L, Weisfelt M, Reitsma JB, Vermeulen M. Clinical features and prognostic factors in adults with bacterial meningitis. *New England Journal of Medicine.* 2004;351(18):1849-1859.

17. Weisfelt M, van de Beek D, Spanjaard L, Reitsma JB, de Gans J. Clinical features, complications, and outcome in adults with pneumococcal meningitis: a prospective case series. *The Lancet Neurology.* 2006;5(2):123-129.

18. Kirkpatrick B, Reeves D, MacGowan A. A review of the clinical presentation, laboratory features, antimicrobial therapy and outcome of 77 episodes of pneumococcal meningitis occurring in children and adults. *Journal of Infection.* 1994;29(2):171-182.

19. Michael B, Sidhu M, Stoeter D, et al. Acute central nervous system infections in adults—a retrospective cohort study in the NHS North West region. *QJM: An International Journal of Medicine.* 2010;103(10):749-758.

20. Gouveia EL, Reis JN, Flannery B, et al. Clinical outcome of pneumococcal meningitis during the emergence of pencillin-resistant Streptococcus pneumoniae: an observational study. *BMC infectious diseases.* 2011;11(1):1-10.

21. Bruyn G, Kremer H, De Marie S, Padberg G, Hermans J, Van Furth R. Clinical evaluation of pneumococcal meningitis in adults over a twelve-year period. *European Journal of Clinical Microbiology and Infectious Diseases.* 1989;8(8):695-700.

22. Ursin Rein P, Jacobsen D, Ormaasen V, Dunlop O. Pneumococcal sepsis requiring mechanical ventilation: cohort study in 38 patients with rapid progression to septic shock. *Acta Anaesthesiologica Scandinavica.* 2018;62(10):1428-1435.

23. Mongardon N, Max A, Bouglé A, et al. Epidemiology and outcome of severe pneumococcal pneumonia admitted to intensive care unit: a multicenter study. *Critical care.* 2012;16(4):1-9.

24. Vergison A. Microbiology of otitis media: a moving target. *Vaccine.* 2008;26:G5-G10.

25. Kaplan DM, Gluck O, Kraus M, Slovik Y. Acute bacterial meningitis caused by acute otitis media in adults: a series of 12 patients. *Ear, Nose & Throat Journal.* 2017;96(1):20.

26. Schwartz LE, Brown RB. Purulent otitis media in adults. *Archives of internal medicine.* 1992;152(11):2301-2304.

27. Lacroix JS, Ricchetti A, Lew D, et al. Symptoms and clinical and radiological signs predicting the presence of pathogenic bacteria in acute rhinosinusitis. *Acta oto-laryngologica.* 2002;122(2):192-196.

28. Hickner JM, Bartlett JG, Besser RE, Gonzales R, Hoffman JR, Sande MA. Principles of appropriate antibiotic use for acute rhinosinusitis in adults: background. *Annals of Internal Medicine.* 2001;134(6):498-505.

29. Hansen JG, Højbjerg T, Rosborg J. Symptoms and signs in culture-proven acute maxillary sinusitis in a general practice population. *APMIS.* 2009;117(10):724-729.

30. Johnson P, Cihon C, Herrington J, Choudhri S. Efficacy and tolerability of moxifloxacin in the treatment of acute bacterial sinusitis caused by penicillin-resistant streptococcus pneumoniae: A pooled analysis. *Clinical Therapeutics.* 2004;26(2):224-231.

31. Penttilä M, Savolainen S, Kiukaanniemi H, Forsblom B, Jousimies-Somer H. Bacterial findings in acute maxillary sinusitis—European study. *Acta Oto-Laryngologica.* 1997;117(sup529):165-168.

32. Benninger M, Brook I, Farrell DJ. Disease severity in acute bacterial rhinosinusitis is greater in patients infected with Streptococcus pneumoniae than in those infected with Haemophilus influenzae. *Otolaryngology--Head and Neck Surgery.* 2006;135(4):523-528.
