# Supplementary material for: Comprehensive review of safety in Experimental Human Pneumococcal Challenge
Source: PLoS One. 2023 May 4;18(5):e0284399. doi: 10.1371/journal.pone.0284399 (PMC10159102; doi:10.1371/journal.pone.0284399)
Supplement: S4 Table — (DOCX) [file pone.0284399.s007.docx]

## **S4 Table: Grading score for adverse events**

| **Grade** | **Grading** | **Definition** |
| --- | --- | --- |
| 1 | Mild | Awareness of symptom but tolerated; transient or mild discomfort; little or no medical intervention required |
| 2 | Moderate | Discomfort enough to cause limitation of usual activity (some assistance may be needed); some medical intervention or therapy required |
| 3 | Severe | Significant interference with daily activity; some assistance usually required; medical intervention/therapy required; hospitalisation possible |
| 4 | Potentially Life threatening | A&E visit or hospitalisation |
