# Supplementary material for: Comprehensive review of safety in Experimental Human Pneumococcal Challenge
Source: PLoS One. 2023 May 4;18(5):e0284399. doi: 10.1371/journal.pone.0284399 (PMC10159102; doi:10.1371/journal.pone.0284399)
Supplement: S1 File — (DOCX) [file pone.0284399.s008.docx]

## **Participant symptom reporting and safety review**

Additional clinical samples (viral/bacterial swabs and blood samples) were obtained when clinically indicated. If a participant contacted the research team out-of-hours, based on the severity and progression of symptoms, an ad-hoc review was performed at the next available time by a member of the clinical team. If their symptoms were particularly concerning the medical team advised review by external emergency care services. Antibiotics, if deemed necessary, were advised based on review by the study clinician.
